# Gender differences in symptomatology, socio-demographic information and quality of life in Spanish population with long COVID condition: a cross-sectional study

**DOI:** 10.3389/fpubh.2024.1355973

**Published:** 2024-03-21

**Authors:** Irene Marcilla-Toribio, Maria Leopolda Moratalla-Cebrián, Blanca Notario-Pacheco, Miguel Angel Escudero-Lopez, Nagore Morales-Cuenca, Maria Martinez-Andres

**Affiliations:** ^1^Universidad de Castilla-La Mancha, Centro de Estudios Sociosanitarios, Cuenca, Spain; ^2^Universidad de Castilla-La Mancha, Grupo de Investigación Health, Gender, Social Determinants, Cuenca, Spain; ^3^Universidad de Castilla- La Mancha, Facultad de Enfermería de Albacete, Albacete, Spain; ^4^Universidad de Castilla-La Mancha, Facultad de Enfermería de Cuenca, Cuenca, Spain

**Keywords:** long COVID, post-acute COVID syndrome, gender perspective, quality of life, symptomatology, epidemiology, public health

## Abstract

**Introduction:**

Long COVID patients experience a decrease in their quality of life due to the symptomatology produced by the disease. It is also important to understand how long COVID affects both men and women. The objective of this study is to examine the impact of long COVID symptomatology on the quality of life of Spanish adults from a gender perspective.

**Methods:**

An observational and cross-sectional study was carried out. Participants were able to complete an online questionnaire using an online platform. A sample of 206 people participated in the study.

**Results:**

The 80.6% of the sample were women with a mean age of 46.51 (±8.28) and the 19.4% were men with a mean age of 48.03 (±9.50). The medium score in the PAC19-QoL test was 141.47 (±24.96) and segmented by gender, 141.65 (±23.95) for women and 140.82 (±28.66) for men. The most common symptoms in women were muscle and joint pain (94.6%), fatigue (94.0%), discomfort (92.2%), difficulty concentrating (91.0%), and memory loss (88.6%). For men the symptoms included muscle and joint pain (97.5%) and fatigue (97.5%) both occupying first position, discomfort (92.0%), difficulty concentrating (90.0%), mood disturbances (90.0%), and memory loss (87.5%). The chi-square test showed statistical significance (*p* < 0.005) for socio-demographic information, quality of life scores, and long COVID symptoms by intensities.

**Conclusion:**

This study shows that there are gender differences in the way that long COVID is experienced.

## Introduction

The COVID-19 pandemic has had a global impact on various spheres of life worldwide (1). In addition to being a public health crisis and causing global economic disruption, this disease has had a significant impact on individual health (1, 2). Cases of incomplete recovery and persistence of symptoms months after the acute phase of the disease have been documented. This is a condition commonly referred to as long COVID (3, 4).

The World Health Organisation (WHO) has defined the term “long COVID” as the persistence of signs, symptoms, or abnormal clinical parameters persisting 3 months following the onset of COVID-19 (with or without a confirmed diagnosis) and with a duration of at least 2 months which cannot be explained by an alternative diagnosis (5). It is estimated that this disease affects 1 in 8 adults, or 12.7%, infected with COVID-19 (6, 7).

Long COVID can affect multiple organ systems and can include very heterogeneous symptoms (3, 8, 9). Although the exact cause of this disease is still not yet fully understood (3, 9, 10), the symptomatology has been well-studied. These more than 200 possible symptoms can be organized into categories such as general, respiratory, cardiac, neurological, psychological, otorhinological, ophthalmological, dermatological and digestive symptoms (3, 9, 11). Fatigue or asthenia, classified as general symptoms, has been reported as the most common symptomatology (3, 9, 11, 12). Other of the most prevalent symptoms reported have been respiratory and neurological symptoms (6, 11, 12). Although long COVID symptomatology has been studied, there is still very little information as regards its impact in terms of intensity (11, 12). Furthermore, long COVID seems to follow a pattern which points to the female gender in their 40s as the group most affected by this disease, however, there is a deficiency in knowledge as regards the differences between symptoms based on gender (3, 13, 14).

Several guidelines have been published on the treatment of long COVID, including rehabilitation and the use of drugs used in similar conditions such as fibromyalgia (10, 15). Additionally, clinical characterization of patients with the illness is essential to provide appropriate therapeutic options (4, 10). However, there is still a significant practical gap that needs to be addressed. Furthermore, to alleviate the burden on individuals with long COVID and the healthcare systems that support them, it is imperative to gain a better understanding of the pathogenesis, risk factors, symptoms and treatment methods of this condition (16).

The effects of an illness usually go beyond its clinical outcome such as mortality and morbidity and encompass the subjective plane in terms of poorer health-related quality of life (17, 18). This disease is known to affect the quality of life of those suffering from long COVID due to the frequency and the burden of persistent symptoms over time (19, 20). In certain circumstances, that situation can be extremely disabling. Unquestionably it is a public health issued that needs to be addressed (3). The importance of assessing the quality of life in people who suffer from this disease is crucial to finding solutions to this disease (3, 19). Emerging evidence suggests that these long-term symptoms have a negative impact on the health-related quality of life of afflicted patients and affect patients’ ability to function in everyday life, including their ability to work (21, 22). Whether persistent symptoms intensities impacts health-related quality of life and if it is differences per gender are still unclear (21, 23).

Currently, there are general validated instruments which assess the quality of life, including EQ-5D, SF-36, and SF-12, but there is likewise a specific tool, thus far, that specifically assesses the quality of life in people suffering from long COVID, the “PAC19-QoL”instrument (24). In addition to being validated in its original language, English, to the best of one’s knowledge, it has likewise been validated in other languages including Spanish (25), Slovak (26), and German (27).

Finally, the prognosis of this disease varies significantly among patients (28). Individual prognosis depends on several factors, including the severity of the initial infection, the presence of comorbidities, and the age and general health of the patient. Although there are currently limited studies on the prognosis and outcome of long COVID, further follow-up studies are necessary to determine the extent of the harm (29, 30).

Based on current knowledge to date, there have been no studies published which examine the impact of long COVID symptoms as regards the quality of life of these patients from a gender perspective. Therefore, the objective of this study is to examine the impact of long COVID symptomatology on the quality of life of Spanish adults from a gender perspective. The secondary objectives were (a) to analyse the influence of socio-demographic variables on quality of life and whether gender-related differences exist and (b) to assess how the intensity of the long COVID symptomatology influences quality of life and the role of gender.

## Methods

### Design

An observational and cross-sectional study was carried out with data collected using an online questionnaire to answer the research questions.

### Participants and data collection

The study used convenience sampling to recruit adults suffering from long COVID in Spain. The researchers invited to participate individuals aged 18 years and above through various Spanish long COVID associations and social media. After receiving information about the study’s objectives and procedures of the study, all the interested participants completed the consent form and then, the online questionnaire via the provided link. Participants were also provided with the contact details (email and telephone number) of the research team to resolve any doubts or problems during the filling of the survey. Finally, 206 people participated in the study. The inclusion criteria were based on the following: to be age 18 and older, have had COVID-19 or suspicions due to compatible symptomatology, have or have had symptomatology over three or more months since the onset of COVID-19 infection, and be able to speak, read, and/or understand Spanish. Individuals with end-stage disease, institutionalization, intellectual disability, dementia, and language barriers were excluded. Participants completed the questionnaire in an online format in the SurveyMonkey online platform account of the University of Castilla-La Mancha between 20 June to 20 July 2022. Security protocols and protection of personal data were upheld.

### Variables and measurement instruments

The variables obtained and the measurement instruments used in the questionnaire for each participant were as follows:

- Sociodemographic information: gender, age, weight, height, marital status, level of education, and dependency in the household.
- Clinical information: COVID-19 and long COVID symptomatology and habits such as drinking alcohol, smoking, sleep problems and comorbidities.
- PAC19-QoL Spanish tool. This questionnaire specifically assesses the quality of life in people with long COVID. This instrument has 5 domains (social, psychological, self-recognition, physical, and work) and 44 items. This enables estimating the impact of long-term COVID on the quality of life of affected patients. Scores range from 0 to 220, with higher scores indicating a lower quality of life.

### Data analysis

The data analysis was performed using the version 28.0 of IBM SPSS statistical software. A descriptive analysis was carried out to provide a profile of participants in the study. For categorical variables, the sample characteristics and responses were presented as frequency and percentage. For continuous data, the variables were reported as mean and standard deviation and/or median/interquartile range. The Kolmmogorov-Smirnov test was used to verify the normality of the variables and the Levene test to verify the homogeneity of variance (Supplementary material). The relationship between sociodemographic characteristics, quartiles of quality of life and symptomatology was stablished using the Chi-Square test. The analysis was considered statistically significant at a *p* value ≤ 0.05.

### Ethical considerations

The study protocol was registered and approved under number 2022/001 by the Clinical Research Ethics Committee from Hospital of Albacete. All research procedures used in this study were established as per the Declaration of Helsinki. All participants provided their consent to participate in the study after being duly information as regards the objectives and procedures.

## Results

This study comprised 206 people with a mean age of 46.81 years (±8.53). Of the total participants 166 were women (80.6%) with a mean age of 46.51 (±8.28) and 40 were men (19.4%) with a mean age of 48.03 (±9.50). The sociodemographic data obtained are shown in Table 1.

**Table 1:** Socio-demographic characteristics of the sample population.

Socio-demographic data	Total group (*n* = 206)	Women (*n* = 166) 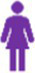	Men (*n* = 40) 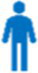
**Age/years**			
Mean ± SD	46.8 ± 8.5	46.5 ± 8.3	48 ± 9.5
No answer	5 (2.4%)	4 (2.4%)	1 (2.5%)
**BMI (Kg/m** ^**2**^ **)**			
Mean ± SD	26.3 ± 5.7	25.9 ± 5.8	27.7 ± 5.5
Underweight	5 (2.4%)	5 (3.0%)	0 (0%)
Normal weight	93 (45.1%)	78 (47%)	15 (37.5%)
Overweight	48 (23.3%)	37 (22.3%)	11 (27.5%)
Obesity	47 (22.8%)	35 (21.1%)	12 (30.0%)
No answer	13 (6.3%)	11 (6.6%)	2 (5.0%)
**Marital status**			
Married	128 (62.1%)	102(61.4%)	26 (65.0%)
Single	46 (22.3%)	36 (21.7%)	10 (25.0%)
Divorced	25 (12.1%)	21 (12.7%)	4 (10.0%)
Other	7 (3.4%)	7 (4.2%)	0 (0%)
**Dependency in the household**			
Yes	29 (14.1%)	21 (12.7%)	8 (20.0%)
No	165 (79.6%)	134 (80.7%)	30 (75%)
No answer	13 (6.3%)	11 (6.6%)	2 (5%)
**Education**			
Primary	10 (4.9%)	7 (4.2%)	3 (7.5%)
Secondary	87 (42.2%)	66 (39.8%)	21 (52.5%)
University or higher	106 (51.5%)	90 (54.2%)	16 (40.0%)
No answer	3 (1.5%)	3 (1.8%)	0 (0%)
**Tobacco**			
Never smoker	102 (49.5%)	83 (50.0%)	19 (47.5%)
Ex-smoker for more than 5 years	61 (29.6%)	48 (28.9%)	13 (32.5%)
Ex-smoker from 1 to 5 years	13 (6.3%)	12 (7.2%)	1 (2.5%)
Sporadic	10 (4.9%)	7 (4.2%)	3 (7.5%)
Regular	10 (4.9%)	8 (4.8%)	2 (5.0%)
**Alcohol**			
Yes	45 (21.8%)	33 (19.9%)	12 (30.0%)
No	160 (77.7%)	132(79.5%)	28 (70.0%)
No answer	1 (0.5%)	1 (0.6%)	0 (0%)
**Sleep problems**			
Yes	160 (77.7%)	129(77.7%)	31 (77.5%)
No	43 (20.9%)	36 (21.7%)	7 (17.5%)
No answer	3 (1.5%)	1 (0.6%)	2 (5.0%)

**Figure 1:**
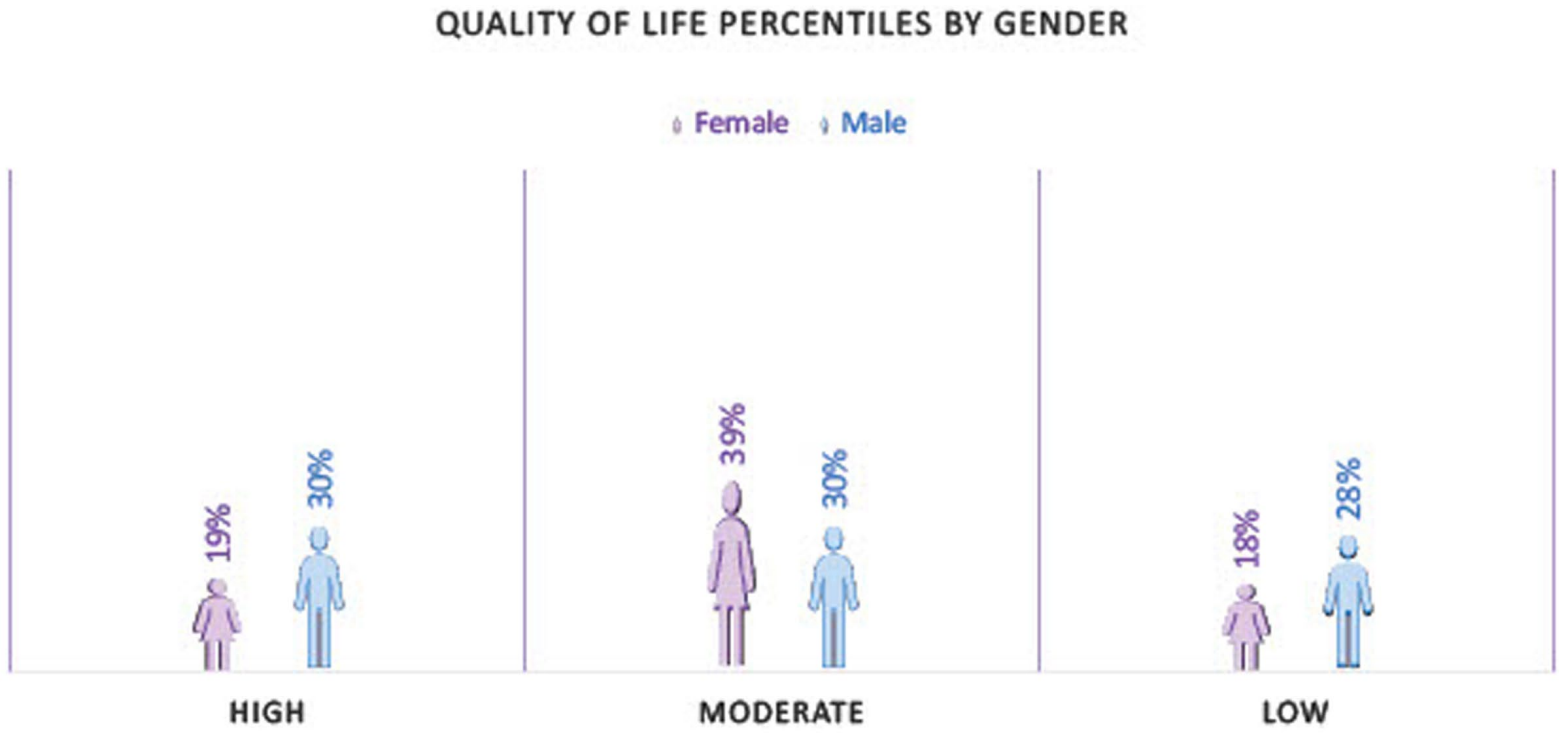
Quality of life percentiles by gender.

To obtain the PAC19-QoL score, 45 questionnaires with unanswered items were considered missing dates. Therefore, 161 questionnaires were scored, 126 (78.3%) were from women, and 35 (21.7%) were from men. The average score in the test was 141.47 (±24.96) and segmented by gender, 141.65 (±23.95) for women and 140.82 (±28.66) for men. A ceiling or floor effect was absent, as any participants scored the minimum (0) or the maximum score (220). Quality of life was also calculated by percentiles. These were classified as 0 to 25th percentile high quality of life, 25th to 75th percentile moderate quality of life, and 75th to 100th percentile low quality of life (Figure 1).

Symptomatology did not follow a normal distribution. The five most common symptoms in female were muscle and joint pain (94.6%), fatigue (94.0%), discomfort (92.2%), difficulty concentrating (91.0%), and memory loss (88.6%). For men, the symptoms included muscle and joint pain (97.5%) and fatigue (97.5%) both occupying first position, discomfort (92.0%), difficulty concentrating (90.0%), mood disturbances (90.0%), and memory loss (87.5%). The frequency of long COVID symptomatology is shown in Table 2.

**Table 2:** Frequency of long COVID symptoms of the sample population by per gender.

Symptoms	TOTAL GROUP (*n* = 206)			WOMEN (*n* = 166) 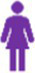			MEN (*n* = 40) 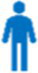		
	*n*	*n* (%)	Rank	*n*	*n* (%)	Rank	*n*	*n* (%)	Rank
Muscle and joint pain	196	95.1%	1	157	94.6%	1	39	97.5%	1
Fatigue	195	94.7%	2	156	94.0%	2	39	97.5%	1
Discomfort	190	92.2%	3	153	92.2%	3	37	92.%	2
Difficulty concentrating	187	90.8%	4	151	91.0%	4	36	90.0%	3
Memory loss	182	88.3%	5	147	88.6%	5	35	87.5%	5
Dyspnoea	175	85.0%	6	141	84.9%	6	34	85.0%	6
Mood disturbances	175	85.0%	6	139	83.7%	8	36	90.0%	4
Headache	173	84.0%	7	142	85.5%	7	31	77.5%	7
Palpitations	160	77.7%	8	131	78.9%	9	29	72.5%	8
Cough	126	61.2%	9	100	60.2%	11	26	65.0%	9
Hair loss	123	59.7%	10	111	66.9%	10	12	30.0%	14
Diarrhea	102	49.5%	11	79	47.6%	12	23	57.5%	10
Olfactory loss	90	43.7%	12	74	44.6%	14	16	40.0%	12
Gustatory loss	88	42.7%	13	75	45.2%	13	13	32.5%	13
Swallowing difficulties	86	41.7%	14	70	42.2%	15	16	40.0%	12
Skin rashes	84	40.8%	15	68	41.0%	16	16	40.0%	12
Conjunctivitis	73	35.4%	16	55	33.1%	17	18	45.0%	11

**Figure 2:**
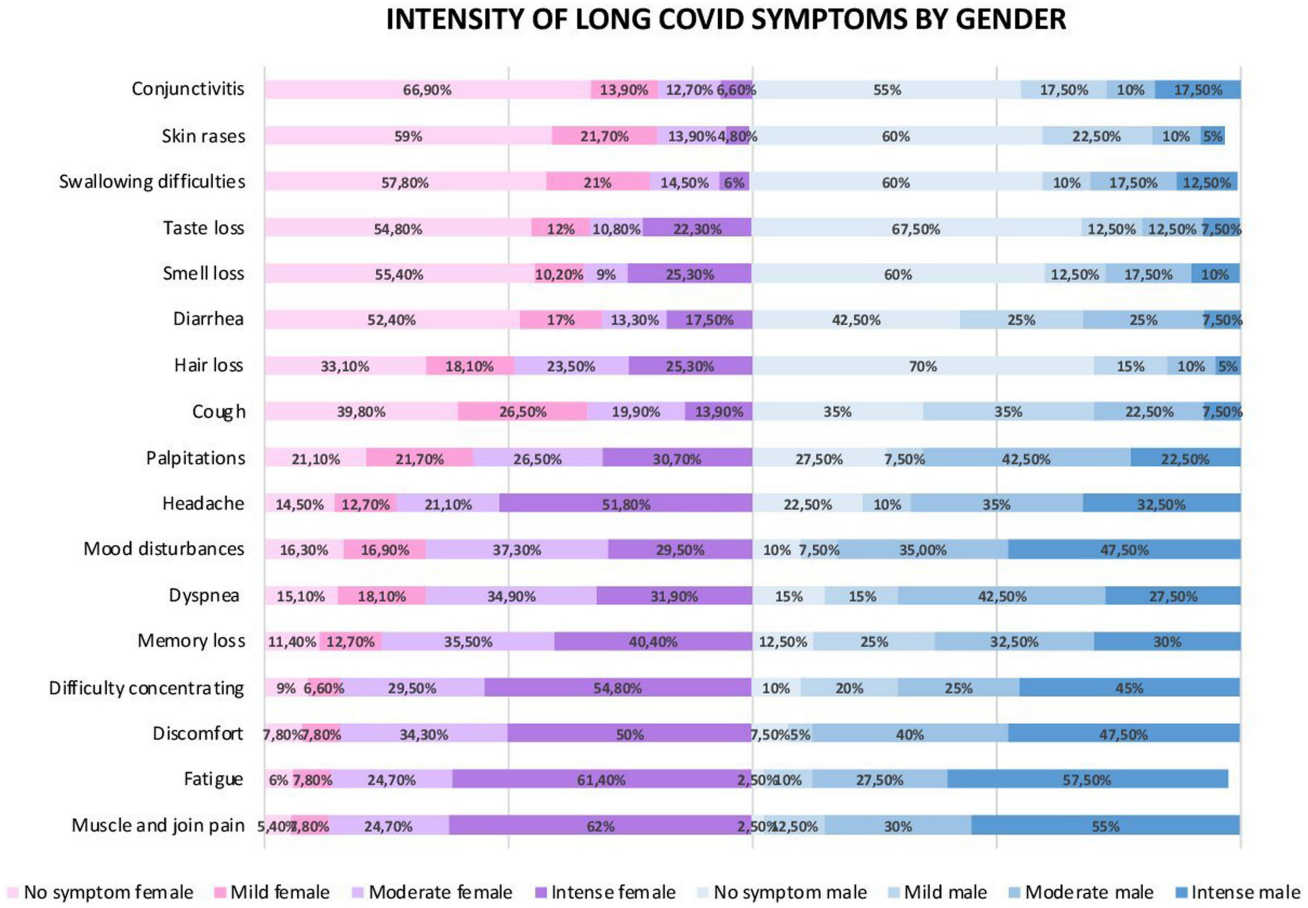
Intensity of long COVID symptoms by gender.

Insofar as symptomatology by intensities are concerned (Figure 2), the three strongest most prevalent symptoms in women were muscle and joint pain (62%), fatigue (61.4%), and concentration difficulties (54.8%). For male, there were fatigue (57.5%), muscle and joint pain (55.0%), and in the third same position were discomfort (47.5%), and mood disturbances (47.5%).

The Chi-square test with the socio-demographic information and the long COVID symptoms as per intensities with the quality-of-life scores of the participants was carried out. In terms of socio-demographic information and quality of life, women showed a *p*-value ≤ 0.05 for the dependency in the household (*p* = 0.045) and sleep problems (*p* = 0.025) variables. Men showed a *p*-value ≤ 0.05 for the overweight (*p* = 0.043), obesity (*p* = 0.032), married (*p* = 0.033), single (*p* = 0.033), primary education (*p* = 0.028), alcohol (*p* = 0.018), and sleep problems (*p* = 0.046) variables. In terms of intensity of symptomatology and quality of life, women showed a *p*-value ≤ 0.05 for all symptoms except for dyspnoea (*p* = 0.078), cough (*p* = 0.109), skin rashes (*p* = 0.104), and conjunctivitis (*p* = 0.090). For men, only taste loss showed a significant *p*-value (*p* = 0.007). For both genders, severe symptom modality was related closely to poorer quality of life score.

Table 3 shows the chi-square test to relate the intensity of the long COVID symptoms and the socio-demographic data. Women showed *p*-value ≤ 0.05 in the BMI category and the fatigue (*p* = 0.046) and diarrhea (*p* = 0.021) symptoms; in the marital status category and the diarrhea (*p* = 0.036), olfactory loss (*p* = 0.026) and difficulty swallowing (*p* = 0.002) symptoms; in the dependency in the household category and the memory loss (*p* = 0.001); in the education category and the diarrhea (*p* = 0.011) symptoms; in the alcohol category and the dyspnoea (*p* = 0.006) symptom; in the sleep problems category and the fatigue (*p* = 0.001), difficulty concentrating (*p* = 0.028), memory loss (*p* = 0.003), palpitations (*p* = 0.028), cough (*p* = 0.005), and difficulty swallowing (*p* = 0.031) symptoms. Men showed *p*-value ≤ 0.05 in the category education and the skin rashes (*p* = 0.036) and conjunctivitis (*p* = 0.046) symptoms; the tobacco category and the cough (*p* = 0.046) symptom; the alcohol category and the discomfort (*p* = 0.023) symptom; the sleep problems category and the difficulty concentrating (*p* = 0.004), and memory loss (*p* = 0.012) symptoms. Distributions and chi-square test of socio-demographic variables and long COVID symptomatology data based on intensity per gender are shown in Tables 4, 5.

**Table 3:** Intensity of long COVID symptoms association with sociodemographic variables per gender.

Long COVID symptoms by intensities	BMI		Marital status		Dependency in the household		Education		Tobacco		Alcohol		Sleep problems	
	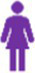	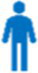	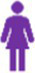	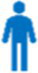	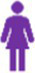	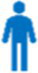	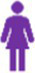	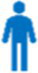	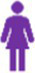	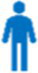	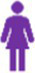	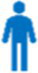	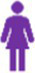	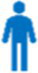
Muscle and joint pain	0.214	0.240	0.052	0.173	0.269	0.499	0.879	0.455	0.192	0.142	0.192	0.186	0.013*	0.569
Fatigue	0.046*	0.803	0.157	0.749	0.116	0.251	0.235	0.492	0.587	0.478	0.060	0.372	0.001*	0.741
Discomfort	0.515	0.525	0.232	0.353	0.217	0.320	0.357	0.373	0.475	0.764	0.213	0.023*	0.264	0.162
Difficulty concentrating	0.560	0.080	0.275	0.935	0.134	0.641	0.518	0.373	0.457	0.517	0.466	0.280	0.028*	0.004*
Memory loss	0.060	0.265	0.118	0.470	0.001*	0.118	0.685	0.395	0.212	0.537	0.474	0.943	0.003*	0.012*
Dyspnoea	0.287	0.097	0.618	0.158	0.595	0.615	0.647	0.692	0.230	0.174	0.006*	0.516	0.101	0.643
Mood disturbances	0.525	0.253	0.242	0.297	0.359	0.899	0.404	0.951	0.915	0.454	0.982	0.465	<0.001*	0.797
Headache	0.903	0.260	0.779	0.889	0.478	0.588	0.151	0.349	0.408	0.764	0.293	0.066	0.070	0.938
Palpitations	0.483	0.284	0.302	0.899	0.500	0.282	0.660	0.321	0.656	0.301	0.157	0.796	0.028*	0.603
Cough	0.076	0.091	0.299	0.214	0.190	0.659	0.613	0.429	0.678	0.046*	0.668	0.139	0.005*	0.116
Hair loss	0.236	0.707	0.587	0.188	0.763	0.425	0.499	0.561	0.167	0.121	0.089	0.166	0.140	0.533
Diarrhea	0.021*	0.659	0.036*	0.333	0.577	0.874	0.363	0.051	0.011*	0.948	0.321	0.179	0.056	0.258
Olfactory loss	0.064	0.837	0.026*	0.277	0.168	0.706	0.792	0.387	0.605	0.409	0.105	0.186	0.133	0.610
Gustatory loss	0.807	0.794	0.446	0.896	0.017*	0.579	0.554	0.311	0.348	0.317	0.055	0.126	0.282	0.266
Swallowing difficulties	0.165	0.764	0.002*	0.801	0.051	0.604	0.972	0.398	0.353	0.373	0.200	0.936	0.031*	0.355
Skin rashes	0.257	0.303	0.150	0.257	0.838	0.433	0.169	0.036*	0.202	0.624	0.063	0.220	0.132	0.210
Conjunctivitis	0.201	0.875	0.447	0.717	0.124	0.965	0.143	0.046*	0.831	0.376	0.212	0.653	0.268	0.370

Chi-square test. *p < 0.05.

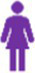
 = women,                                  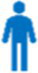 = men, BMI, Body mass.

**Table 4:** Distribution of women data of socio-demographic and long COVID symptomatology variables based on intensity.

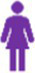	High BMI			Married			Dependency in the household			Education			Tobacco			Alcohol			Sleep problems		
	No	Yes	*p*	No	Yes	*p*	No	Yes	*p*	Basic	High	*p*	No	Yes	*p*	No	Yes	*p*	No	Yes	*p*
**Muscle and joint pain**			0.214			0.052			0.269			0.879			0.192			0.192			**0**.013*
No symptom	3	5		3	6		9	0		4	5		2	7		5	4		5	4	
Mild	9	4		9	4		11	1		6	7		1	12		11	2		5	8	
Moderate	23	13		11	30		34	3		21	20		10	31		31	10		10	30	
Severe	48	50		41	62		80	17		45	58		35	68		85	17		16	87	
**Fatigue**			0.046*			0.157			0.116			0.235			0.587			0.060			0.001*
No symptom	7	1		6	4		9	0		3	7		1	9		5	5		6	4	
Mild	6	7		4	9		11	2		5	8		4	9		9	4		6	7	
Moderate	25	13		11	30		28	9		24	17		13	28		34	7		9	32	
Severe	45	51		43	59		86	10		44	58		30	72		84	17		15	86	
**Discomfort**			0.515			0.232			0.217			0.357			0.475			0.213			0.264
No symptom	8	4		3	10		12	0		3	10		2	11		8	5		5	8	
Mild	7	6		8	5		11	0		7	6		3	10		9	4		4	9	
Moderate	31	22		21	36		48	8		26	31		15	42		48	9		13	43	
Severe	37	40		32	51		63	13		40	43		28	55		67	15		14	69	
**Difficulty concentrating**			0.560			0.275			0.134			0.518			0.457			0.466			0.028*
No symptom	7	8		5	10		14	1		8	7		3	12		10	5		7	8	
Mild	7	4		4	7		11	0		3	8		4	7		8	2		3	8	
Moderate	22	25		14	35		42	4		21	28		11	38		38	11		13	36	
Severe	47	35		41	50		67	16		44	47		30	61		76	15		13	77	
**Memory loss**			0.060			.118			0.001*			0.685			0.212			0.474			0.003*
No symptom	12	6		7	12		16	3		7	12		3	16		13	6		10	9	
Mild	11	7		7	14		20	0		8	13		5	16		15	5		3	18	
Moderate	22	34		17	42		52	2		29	30		15	44		48	11		14	45	
Severe	38	25		33	34		46	16		32	35		25	42		56	11		9	57	
**Dyspnoea**			0.287			0.618			0.595			0.647			0.230			0.006*			0.101
No symptom	13	9		10	15		21	2		13	12		4	21		15	10		10	15	
Mild	19	10		10	20		23	5		16	14		10	20		21	9		7	23	
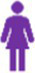	High BMI			Married			Dependency in the household			Education			Tobacco			Alcohol			Sleep problems		
	No	Yes	*p*	No	Yes	*p*	No	Yes	*p*	Basic	High	*p*	No	Yes	*p*	No	Yes	*p*	No	Yes	*p*
Moderate	29	25		20	38		45	9		24	34		21	37		48	9		10	47	
Severe	24	28		24	39		45	5		23	30		13	40		48	5		9	44	
**Mood disturbances**			0.525			0.242			0.359			0.404			0.915			0.982			<0.001*
No symptom	16	10		15	12		22	2		10	17		7	20		21	6		13	14	
Mild	11	13		11	17		23	4		13	15		8	20		23	5		6	21	
Moderate	34	25		21	41		46	11		26	36		17	45		49	12		14	48	
Severe	22	24		17	32		43	4		27	22		16	33		39	10		3	46	
**Headache**			0.903			0.779			0.478			0.151			0.408			0.293			0.070
No symptom	13	9		9	15		21	1		12	12		4	20		16	8		8	16	
Mild	10	11		9	12		18	2		5	16		6	15		16	5		7	14	
Moderate	17	15		11	24		27	6		19	16		9	26		28	6		9	25	
Severe	43	37		35	51		68	12		40	46		29	57		72	14		12	74	
**Palpitations**			0.483			0.302			0.500			0.660			0.656			0.157			0.028*
No symptom	20	11		15	20		27	4		18	17		8	27		25	10		13	22	
Mild	16	19		11	25		28	6		14	22		9	27		26	10		9	27	
Moderate	21	20		14	30		39	3		22	22		15	29		37	6		4	39	
Severe	26	22		24	27		40	8		22	29		16	35		44	7		10	41	
**Cough**			0.076			0.299			0.190			0.613			0.678			0.668			0.005*
No symptom	39	22		20	46		56	5		33	33		21	45		50	16		23	43	
Mild	19	25		19	25		32	9		19	25		10	34		36	8		6	37	
Moderate	17	12		16	17		28	3		16	17		9	24		26	6		2	31	
Severe	8	13		9	14		18	4		8	15		8	15		20	3		5	18	
**Hair loss**			0.236			0.587			0.763			0.499			0.167			0.089			0.140
No symptom	24	28		20	35		46	5		29	26		11	44		44	10		17	37	
Mild	13	16		9	21		24	4		12	18		12	18		20	10		6	24	
Moderate	22	13		16	23		31	5		15	24		10	29		30	9		8	31	
Severe	24	15		19	23		33	7		20	22		15	27		38	4		5	37	
**Diarrhea**			0.021*			0.036*			0.577			0.363			0.011*			0.321			0.056
No symptom	52	30		25	62		73	9		41	46		20	67		64	22		26	61	
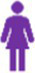	High BMI			Married			Dependency in the household			Education			Tobacco			Alcohol			Sleep problems		
	No	Yes	*p*	No	Yes	*p*	No	Yes	*p*	Basic	High	*p*	No	Yes	*p*	No	Yes	*p*	No	Yes	*p*
Mild	15	12		14	14		21	3		9	19		5	23		24	4		3	24	
Moderate	7	13		9	13		18	3		10	12		8	14		19	3		4	18	
Severe	9	17		16	13		22	6		16	13		15	14		25	4		3	26	
**Olfactory loss**			0.064			0.026*			0.168			0.792			0.605			0.105			0.133
No symptom	45	40		31	61		73	12		43	49		26	66		68	24		23	68	
Mild	4	12		5	12		12	4		8	9		7	10		16	1		0	17	
Moderate	9	6		11	4		11	3		5	10		3	12		14	1		4	11	
Severe	25	14		17	25		38	2		20	22		12	30		34	7		9	33	
**Gustatory loss**			0.807			0.446			0.017*			0.554			0.348			0.055			0.282
No symptom	45	40		30	61		77	12		41	50		28	63		68	23		22	68	
Mild	9	9		9	11		11	6		8	12		7	13		20	0		1	19	
Moderate	8	9		8	10		13	2		11	7		2	16		16	2		4	14	
Severe	21	14		17	20		33	1		16	21		11	26		18	8		9	28	
**Swallowing difficulties**			0.165			0.002*			0.051			0.972			0.353			0.200			0.031*
No symptom	54	35		29	67		82	8		45	51		26	70		71	24		28	68	
Mild	14	18		12	23		28	4		15	20		8	27		32	3		2	32	
Moderate	11	13		16	8		17	6		11	13		10	14		20	4		5	19	
Severe	3	6		7	3		6	3		5	5		4	6		8	2		1	9	
**Skin rashes**			0.257			0.150			0.838			0.169			0.202			0.063			0.132
No symptom	56	37		34	64		82	11		48	50		25	73		72	25		27	71	
Mild	15	18		14	22		28	5		11	25		10	26		33	3		4	31	
Moderate	8	12		10	13		17	4		12	11		11	12		19	4		3	20	
Severe	4	4		6	2		6	1		5	3		2	6		8	0		1	7	
**Conjunctivitis**			0.201			0.447			0.124			0.143			0.831			0.212			0.268
No symptom	62	44		43	68		93	11		52	59		30	81		83	27		28	82	
Mild	7	14		9	14		18	3		6	17		8	15		21	2		3	20	
Moderate	10	10		10	11		16	3		11	10		6	15		18	3		2	19	
Severe	4	4		2	9		7	4		7	4		4	7		10	1		3	8	

Chi-square test. *p < 0.05.

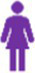
 = women,                                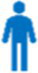 = men, BMI, Body mass. Bold values = Chi-square test. *p < 0.05 (this is written in the legend). As there are so many numbers, it was decided to put the symbol * for *p* < 0.05 and to mark them in bold to make them more visual.

**Table 5:** Distribution of men data of socio-demographic and long COVID symptomatology variables based on intensity.

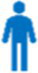	High BMI			Married			Dependency in the household			Education			Tobacco			Alcohol			Sleep problems		
	No	Yes	*p*	No	Yes	*p*	No	Yes	*p*	Basic	High	*p*	No	Yes	*p*	No	Yes	*p*	No	Yes	*p*
**Muscle and joint pain**			0.240			0.173			0.499			0.455			0.142			0.186			0.569
No symptom	0	1		0	1		1	0		0	1		0	1		1	0		0	1	
Mild	3	2		1	4		5	0		3	2		1	4		2	3		2	3	
Moderate	2	9		2	10		9	2		6	6		7	5		7	5		2	9	
Severe	10	11		11	11		15	6		15	7		5	17		18	4		3	18	
**Fatigue**			0.803			0.749			0.251			0.492			0.478			0.372			0.741
No symptom	0	1		0	1		1	0		0	1		0	1		1	0		0	1	
Mild	2	2		1	3		4	0		2	2		2	2		2	2		1	3	
Moderate	5	6		5	6		10	1		8	3		2	9		6	5		3	8	
Severe	8	13		8	15		14	7		14	9		9	14		18	5		3	19	
**Discomfort**			0.525			0.353			0.320			0.373			0.764			0.023*			0.162
No symptom	0	3		0	3		3	0		1	2		1	2		2	1		0	3	
Mild	1	1		0	2		2	0		1	1		0	2		2	0		1	0	
Moderate	6	9		7	9		13	2		8	8		6	10		7	9		3	13	
Severe	8	10		7	12		12	6		14	5		6	13		17	2		3	15	
**Difficulty concentrating**			0.080			0.935			0.641			0.373			0.517			0.280			0.004*
No symptom	3	1		1	3		3	1		3	1		0	4		3	1		0	4	
Mild	1	7		3	5		6	1		4	4		3	5		6	2		5	3	
Moderate	6	4		3	7		9	1		8	2		4	6		9	1		1	7	
Severe	5	11		7	11		12	5		9	9		6	12		10	8		1	17	
**Memory loss**			0.265			0.470			0.118			0.395			0.537			0.943			0.012*
No symptom	3	23		1	4		4	1		4	1		1	4		3	2		0	5	
Mild	3	7		5	5		8	1		4	6		2	8		7	3		5	4	
Moderate	7	6		3	10		12	1		9	4		6	7		9	4		1	11	
Severe	2	8		5	7		6	5		7	5		4	8		9	3		1	11	
**Dyspnoea**			0.097			0.158			0.615			0.692			0.174			0.516			0.643
No symptom	3	3		3	3		5	1		3	3		1	5		5	1		0	6	
Mild	1	5		0	6		4	2		4	2		4	2		4	2		1	4	
Moderate	9	6		8	9		14	2		9	8		6	11		10	7		4	13	
Severe	2	9		3	8		7	3		8	3		2	9		9	2		2	8	
**Mood disturbances**			0.253			0.297			0.899			0.951			0.454			0.465	1	3	0.797
No symptom	3	1		0	4		3	1		2	2		0	4		3	1		0	3	
Mild	0	3		1	2		2	0		2	1		1	2		3	0		3	10	
Moderate	5	8		4	10		11	3		8	6		6	8		8	6		3	15	
Severe	7	11		9	10		14	4		12	7		6	13		14	5				
**Headache**			0.260			0.889			0.588			0.349			0.764			0.066			0.938
No symptom	4	5		4	5		8	1		7	2		3	6		6	3		1	7	
Mild	0	4		1	3		3	0		1	3		2	2		1	3		1	3	
Moderate	7	6		5	9		10	4		8	6		5	9		9	5		3	11	
Severe	4	8		4	9		9	3		8	5		3	10		12	1		2	10	
**Palpitations**			0.284			0.899			0.282			0.321			0.301			0.796			0.603
No symptom	4	7		3	8		8	3		9	2		3	8		9	2		3	8	
Mild	0	3		1	2		1	1		1	2		1	2		2	1		0	3	
Moderate	6	10		7	10		12	4		9	8		8	9		11	6		2	14	
Severe	5	3		3	6		9	0		5	4		1	8		6	3		2	6	
**Cough**			0.091			0.214			0.659			0.429			0.046*			0.139			0.116
No symptom	8	6		7	7		11	2		9	5		1	13		10	4		1	12	
Mild	6	7		3	11		11	3		7	7		8	6		7	7		3	11	
Moderate	1	7		2	7		6	3		7	2		3	6		8	1		1	7	
Severe	0	3		2	1		2	0		1	2		1	2		3	0		2	1	
**Hair loss**			0.707			0.188			0.425			0.561			0.121			0.166			0.533
No symptom	10	17		10	18		20	7		16	12		7	21		21	7		6	22	
Mild	3	2		1	5		6	0		3	3		3	3		2	4		0	5	
Moderate	1	3		1	3		2	1		3	1		3	1		3	1		1	2	
Severe	1	1		2	0		2	0		2	0		0	2		2	0		0	2	
**Diarrhea**			0.659			0.333			0.874			0.051			0.948			0.179			0.258
No symptom	5	12		4	13		12	4		14	3		5	12		14	3		3	13	
Mild	4	4		3	7		8	2		3	7		4	6		5	5		0	10	
Moderate	5	5		5	5		8	2		5	5		3	7		6	4		3	6	
Severe	1	2		2	1		2	0		2	1		1	2		3	0		1	2	
**Olfactory loss**			0.837			0.277			0.706			0.387			0.409			0.186			0.610
No symptom	9	15		7	17		18	6		13	11		8	16		16	8		5	18	
Mild	1	3		1	4		3	1		3	2		3	2		2	3		0	5	
Moderate	3	3		3	4		5	1		4	3		1	6		6	1		1	6	
Severe	2	2		3	1		4	0		4	0		1	3		4	0		1	2	
**Gustatory loss**			0.794			0.896			0.579			0.311			0.317			0.126			0.266
No symptom	12	15		10	17		21	5		14	13		9	18		18	9		6	20	
Mild	1	3		1	4		3	1		3	2		3	2		2	3		0	5	
Moderate	1	3		2	3		3	2		4	1		1	4		5	0		0	5	
Severe	1	2		1	2		3	0		3	0		0	3		3	0		1	1	
**Swallowing difficulties**			0.764			0.801			0.604			0.398			0.373			0.936			0.355
No symptom	11	13		7	17		18	5		14	10		9	15		16	8		4	18	
Mild	1	2		2	2		2	1		2	2		1	3		3	1		1	3	
Moderate	2	5		3	4		5	2		6	1		3	4		5	2		0	7	
Severe	1	3		2	3		5	0		2	3		0	5		4	1		2	3	
**Skin rashes**			0.303			0.257			0.433			0.036*			0.624			0.220			0.210
No symptom	8	16		9	15		19	4		16	8		6	18		18	6		6	16	
Mild	5	3		1	8		7	2		4	5		4	5		4	5		0	9	
Moderate	2	2		2	2		2	2		4	0		2	2		3	1		0	4	
Severe	0	2		2	1		2	0		0	3		1	2		3	0		1	2	
**Conjunctivitis**			0.875			0.717			0.965			0.046*			0.376			0.653			0.370
No symptom	9	13		6	16		17	5		11	11		7	15		15	7		5	16	
Mild	3	3		3	4		4	1		5	2		4	3		5	2		0	6	
Moderate	1	2		2	2		3	1		1	3		1	3		2	2		0	4	
Severe	2	5		3	4		6	1		7	0		1	6		6	1		2	5	

Chi-square test. *p < 0.05.

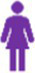
 = women,                                 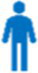 = men, BMI, Body mass. Bold values = Chi-square test. *p < 0.05 (this is written in the legend). As there are so many numbers, it was decided to put the symbol * for *p* < 0.05 and to mark them in bold to make them more visual.

## Discussion

The main objective of this study was to examine the impact of long COVID symptomatology on the quality of life of Spanish adults from a gender perspective. Socio-demographic variables and intensity of the long COVID symptoms were significantly related with the quality-of-life scores obtained. Likewise, significant differences per gender were identified.

The findings suggest that long COVID is more prevalent in females. Several studies on individuals with long COVID condition have found that over half of the sample population is female, which is consistent with the results of our cross-sectional study (13, 31, 32). In addition, this trend is also consistent with the average age of those affected, which is around 40 years old. These findings align with the results previously reported in other studies on the subject (13, 33, 34).

Other similarities related with the more prevalent symptoms were compared with the findings obtained by Anaya et al. (35) and Aiyegbusi et al. (36) in which are fatigue and muscle and joint pain are the most common symptoms. In terms of quality of life, this element was reported to be affected by the disease in other studies (20, 37). Quality of life of long COVID patients was measured from different perspectives in two studies. The quantitative study (20), using generic quality of life scales as EQ-5D-5L, and the qualitative study (37). Although different methodologies were used, both concluded that long COVID influences the quality of life of those with the disease hindering same. It is important to note that, although they are distinct entities, long COVID shares some similarities in terms of symptoms and challenges in diagnosis and treatment with other conditions, such as fibromyalgia (15, 38, 39). Furthermore, studies have also shown that long COVID, fibromyalgia and chronic fatigue syndrome are more prevalent among females (40, 41).

Analyzing gender in health has proven to be crucial due to the differences between men and women (42). These gender differences occur not only in acute but similarly in chronic diseases (42, 43). In this regard, the study has likewise demonstrated differences between both genders in relation to long COVID symptomatology. Male participants in the study were not only more likely to have mood disturbances the female participants but similarly experienced that symptom more acutely than the latter. This can be explained by the fact that age plays a role on resilience, with middle-aged women being more resilient than men (44, 45). As a matter of fact low resilience has been shown to be related to the development of mood disorders (46). Nevertheless, more studies are required to analyse resilience in chronic diseases from a gender perspective.

In terms of quality of life, this study showed statistically significant relationship between the presence of a dependent person in the household and the female gender. Women in nurturing roles often experience a decrease in their quality of life as compared to men. These disparities are due to a number of socio-cultural and economic factors that have been comprehensively discussed in academic literature (47, 48). These factors have a substantial impact on women’s quality of life, as women assume an unequal burden of care responsibilities in the domestic sphere as compared to their male counterparts (48–50).

Another key point of the findings is that women had a higher number of symptoms which were closely related to a lower quality of life. Although it is important to take into account that pain perception is a complex and multifactorial phenomenon, certain studies suggest that women may have a greater sensitivity to pain as compared to men (51, 52). Furthermore, women are known to have a higher prevalence of chronic pain conditions, such as fibromyalgia (51, 53). Coupled with pain, whether chronic, acute or disease-related, can have a significant negative impact on a person’s quality of life (54).

It is likewise remarkable how sleep problems affect not only the symptoms but also the intensity with which these are experienced in both genders. Sleep plays a crucial role in a person’s health and well-being, and sleep deprivation can weaken the body and worsen the symptoms of a disease. In regard to this, being in pain likewise hinders the possibility of having good quality sleep (55). Sleep deprivation can increase stress levels and reduce pain tolerance (56). This fact can exacerbate the symptoms of an illness, especially if this entails chronic pain (55). Continuing with that series of factors which produces a more intense symptomatology, smoking was identified to be a condition that exacerbated long COVID symptomatology. These findings are consistent with those of a study carried out on patients with fibromyalgia which concluded that tobacco was closely related to more severe symptomatology (57).

Elsewhere, this study identified that both men and women who had a dependent in the household were more likely to have a household dependency experienced less severe symptoms of their illnesses than those who did not have this condition at home. The Hamptom & Newcomb study (58) has demonstrated that informal caregivers may have reduced perception of pain due to psychological factors such as an increased ability to handle stress. Furthermore, another important finding of the study as regards the intensity of symptomatology was that for both genders non-drinkers experienced the most intense symptoms. Although the reasons for this are unclear, the findings are consistent with the Kim et al. (59) study which concluded that low and moderate alcohol consumption is associated with a decrease in fibromyalgia symptoms.

### Strengths and limitations

Insofar as the limitation of the present study are concerned, the following were identified. Firstly, the inherent factors of the disease hindered data collection. The cognitive problems associated with long COVID disease including difficulty concentrating and mental fogginess, was prejudicial to the sample numbers as many participants started the questionnaire but dropped out halfway through. Even so, participation was facilitated to the extent practicable by sending notifications as regards the status of the questionnaires and reminders to complete same. Secondly, research has been undertaken from a cross-sectional perspective. A follow-up study yield further information as regards the people participating in that study and the behavior of the disease over time. Likewise, causal relationships could be obtained. Moreover, studies with a higher sample and homogeneous number of men and women should be carried out. Nevertheless, difficulties may be experienced in this homogeneity as this disease seems to affect more women (3, 33, 34). Furthermore, other studies have likewise had more women in the sample (33, 34, 60). It is also important to note that factors as specifying the wave of the pandemic and the variant of the COVID-19 virus, may impact the symptoms of the disease and should therefore, be included in future studies. Finally, it should be noted as a strong point of this study is that it is the first of its kind to take into account the intensities of symptomatology and gender differences, which is of fundamental importance in health science research.

## Conclusion

The findings of this research show that there are gender differences in the way that long COVID is experienced. The most acute symptoms experienced by females are muscle and joint pain, fatigue, and concentration difficulties. In males, the most acute symptoms are fatigue, muscle and joint pain, discomfort, and mood disturbances. Undertaking a gender-sensitive study is important because it helps to understand and address gender inequalities and promote gender equality. The findings suggest future lines of research to design more effective, specific, and personalized care for this emerging disease. Furthermore, longitudinal studies should be carried out to explore the risk factors closely related to long COVID and its relationship to quality of life. Finally, exploring differences in the experience of this disease between different groups of people, such as different ethnic groups or people with pre-existing conditions, should likewise be carried out.

## Data availability statement

The datasets presented in this article are not readily available because the database is part of a cohort study. Requests to access the datasets should be directed to MM-A, maria.martinezandres@uclm.es.

## Ethics statement

The studies involving humans were approved by the Clinical Research Ethics Committee from Hospital of Albacete (number 2022/001). The studies were conducted in accordance with the local legislation and institutional requirements. The participants provided their written informed consent to participate in this study.

## Author contributions

IM-T: Writing – original draft, Writing – review & editing, Conceptualization, Formal analysis, Methodology. MM-C: Writing – original draft, Writing – review & editing, Conceptualization, Methodology. BN-P: Writing – original draft, Writing – review & editing, Formal analysis, Methodology, Resources. ME-L: Writing – original draft, Writing – review & editing. NM-C: Writing – original draft, Writing – review & editing. MM-A: Writing – original draft, Writing – review & editing, Conceptualization, Funding acquisition, Methodology, Resources, Supervision.
