# Supplementary material for: Gender differences in symptomatology, socio-demographic information and quality of life in Spanish population with long COVID condition: a cross-sectional study
Source: Front Public Health. 2024 Mar 21;12:1355973. doi: 10.3389/fpubh.2024.1355973 (PMC10991816; doi:10.3389/fpubh.2024.1355973)
Supplement: Supplementary file 1 [file Table_1.DOCX]

**SUPPLEMENTARY MATERIAL**

**Table S1:** Normality tests of the sociodemographic variables.

**KOLMOGOROV-SMIRNOV^a^**

| GENDER | | STATISTIC | gl | SIG. |
| --- | --- | --- | --- | --- |
| Age | Male | 0.126 | 34 | 0.191 |
|  | Female | 0.051 | 141 | 0.200 |
| BMI* | Male | 0.383 | 34 | <0.001 |
|  | Female | 0.361 | 141 | <0.001 |
| Marital status | Male | 0.429 | 34 | <0.001 |
|  | Female | 0.408 | 141 | <0.001 |
| Household dependency | Male | 0.472 | 34 | <0.001 |
|  | Female | 0.513 | 141 | <0.001 |
| Educational level | Male | 0.414 | 34 | <0.001 |
|  | Female | 0.383 | 141 | <0.001 |
| Tobacco  smoking | Male | 0.429 | 34 | <0.001 |
|  | Female | 0.440 | 141 | <0.001 |
| Alcohol drinking | Male | 0.429 | 34 | <0.001 |
|  | Female | 0.485 | 141 | <0.001 |
| Sleep | Male | 0.486 | 34 | <0.001 |
|  | Female | 0.485 | 141 | <0.001 |

*BMI, body mass index.

^a^ Lilliefors’ significance correction.

**Table S2**: Homogeneity of variance test of the sociodemographic variables.

|  | | LEVENE STATISTIC | GL1 | GL2 | SIG |
| --- | --- | --- | --- | --- | --- |
| Age | Based on the average | 0.679 | 1 | 173 | 0.411 |
|  | Based on the median | 0.448 | 1 | 173 | 0.504 |
|  | Based on the median and with adjusted gl | 0.448 | 1 | 160.995 | 0.504 |
|  | Based on the trimmed mean | 0.536 | 1 | 173 | 0.465 |
| BMI* | Based on the average | 1.584 | 1 | 173 | 0.210 |
|  | Based on the median | 0.265 | 1 | 173 | 0.607 |
|  | Based on the median and with adjusted gl | 0.265 | 1 | 173.000 | 0-607 |
|  | Based on the trimmed mean | 1.584 | 1 | 173 | 0.210 |
| Marital  Status | Based on the average | 1.154 | 1 | 173 | 0.284 |
|  | Based on the median | 0.241 | 1 | 173 | 0.624 |
|  | Based on the median and with adjusted gl | 0.241 | 1 | 172.690 | 0.624 |
|  | Based on the trimmed mean | 1.154 | 1 | 173 | 0.284 |
| Household dependency | Based on the average | 5.093 | 1 | 173 | 0.025 |
|  | Based on the median | 1.473 | 1 | 173 | 0.226 |
|  | Based on the median and with adjusted gl | 1.473 | 1 | 168.493 | 0.227 |
|  | Based on the trimmed mean | 5.093 | 1 | 173 | 0.025 |
| Educational level | Based on the average | 2.682 | 1 | 173 | 0.109 |
|  | Based on the median | 0.483 | 1 | 173 | 0.488 |
|  | Based on the median and with adjusted gl | 0.483 | 1 | 172.957 | 0.488 |
|  | Based on the trimmed mean | 2.682 | 1 | 173 | 0.103 |
| Tobacco smoking | Based on the average | 0.165 | 1 | 173 | 0.685 |
|  | Based on the median | 0.044 | 1 | 173 | 0.835 |
|  | Based on the median and with adjusted gl | 0.044 | 1 | 172.917 | 0.835 |
|  | Based on the trimmed mean | 0.165 | 1 | 173 | 0.685 |
| Alcohol drinking | Based on the average | 5.734 | 1 | 173 | 0.018 |
|  | Based on the median | 1.872 | 1 | 173 | 0.173 |
|  | Based on the median and with adjusted gl | 1.872 | 1 | 170.368 | 0.173 |
|  | Based on the trimmed mean | 5.734 | 1 | 173 | 0.018 |
| Sleep | Based on the average | 0.031 | 1 | 173 | 0.860 |
|  | Based on the median | 0.008 | 1 | 173 | 0.930 |
|  | Based on the median and with adjusted gl | 0.008 | 1 | 173.000 | 0.930 |
|  | Based on the trimmed mean | 0.031 | 1 | 173 | 0.860 |

*BMI, body mass index.

**Table S3**: Normality tests of the symptomatology variables.

**KOLMOGOROV-SMIRNOV^a^**

| GENDER | | STATISTIC | gl | SIG. |
| --- | --- | --- | --- | --- |
| Discomfort | Male | 0.535 | 39 | 0.191 |
|  | Female | 0.538 | 164 | 0.200 |
| Fatigue | Male | 0.538 | 39 | <0.001 |
|  | Female | 0.539 | 164 | <0.001 |
| Muscle and joint pain | Male | 0.538 | 39 | <0.001 |
|  | Female | 0.540 | 164 | <0.001 |
| Cough | Male | 0.424 | 39 | <0.001 |
|  | Female | 0.397 | 164 | <0.001 |
| Dyspnoea | Male | 0.509 | 39 | <0.001 |
|  | Female | 0.514 | 164 | <0.001 |
| Diahrroea | Male | 0.371 | 39 | <0.001 |
|  | Female | 0.350 | 164 | <0.001 |
| Skin rashes | Male | 0.385 | 39 | <0.001 |
|  | Female | 0.388 | 164 | <0.001 |
| Hair loss | Male | 0.450 | 39 | <0.001 |
|  | Female | 0.425 | 164 | <0.001 |
| Headache | Male | 0.487 | 39 | <0.001 |
|  | Female | 0.514 | 164 | <0.001 |
| Difficulty concentrating | Male | 0.528 | 39 | <0.001 |
|  | Female | 0.533 | 164 | <0.001 |
| Loss of taste | Male | 0.424 | 39 | <0.001 |
|  | Female | 0.366 | 164 | <0.001 |
| Loss of smell | Male | 0.385 | 39 | <0.001 |
|  | Female | 0.369 | 164 | <0.001 |
| Mood disturbance | Male | 0.528 | 39 | <0.001 |
|  | Female | 0.509 | 164 | <0.001 |
| Palpitations | Male | 0.450 | 39 | <0.001 |
|  | Female | 0.488 | 164 | <0.001 |
| Difficulty swallowing | Male | 0.385 | 39 | <0.001 |
|  | Female | 0.382 | 164 | <0.001 |
| Conjuctivitis | Male | 0.358 | 39 | <0.001 |
|  | Female | 0.425 | 164 | <0.001 |

^a^ Lilliefors’ significance correction.

**Table S4**: Homogeneity of variance test of the symptomatology variables.

|  | | LEVENE STATISTIC | GL1 | GL2 | SIG |
| --- | --- | --- | --- | --- | --- |
| Discomfort | Based on the average | 0.186 | 1 | 201 | 0.667 |
|  | Based on the median | 0.047 | 1 | 201 | 0.828 |
|  | Based on the median and with adjusted gl | 0.047 | 1 | 200.282 | 0.828 |
|  | Based on the trimmed mean | 0.186 | 1 | 201 | 0.667 |
| Fatigue | Based on the average | 3.251 | 1 | 201 | 0.073 |
|  | Based on the median | 0.763 | 1 | 201 | 0.383 |
|  | Based on the median and with adjusted gl | 0.763 | 1 | 189.815 | 0.384 |
|  | Based on the trimmed mean | 1.354 | 1 | 201 | 0.246 |
| Muscle and joint pain | Based on the average | 2.407 | 1 | 201 | 0.123 |
|  | Based on the median | 0.571 | 1 | 201 | 0.451 |
|  | Based on the median and with adjusted gl | 0.571 | 1 | 191.692 | 0.451 |
|  | Based on the trimmed mean | 0.790 | 1 | 201 | 0.375 |
| Cough | Based on the average | 2.205 | 1 | 201 | 0.139 |
|  | Based on the median | 0.430 | 1 | 201 | 0.513 |
|  | Based on the median and with adjusted gl | 0.430 | 1 | 200.930 | 0.513 |
|  | Based on the trimmed mean | 2.205 | 1 | 201 | 0.139 |
| Dyspnoea | Based on the average | 0.055 | 1 | 201 | 0.815 |
|  | Based on the median | 0.014 | 1 | 201 | 0.906 |
|  | Based on the median and with adjusted gl | 0.014 | 1 | 200.288 | 0.906 |
|  | Based on the trimmed mean | 0.055 | 1 | 201 | 0.815 |
| Diahrroea | Based on the average | 1.699 | 1 | 201 | 0.194 |
|  | Based on the median | 0.263 | 1 | 201 | 0.609 |
|  | Based on the median and with adjusted gl | 0.263 | 1 | 200.999 | 0.609 |
|  | Based on the trimmed mean | 1.699 | 1 | 201 | 0.194 |
| Skin rashes | Based on the average | 0.002 | 1 | 201 | 0.969 |
|  | Based on the median | 0.000 | 1 | 201 | 0.984 |
|  | Based on the median and with adjusted gl | 0.000 | 1 | 200.986 | 0.984 |
|  | Based on the trimmed mean | 0.002 | 1 | 201 | 0.969 |
| Hair loss | Based on the average | 1.929 | 1 | 201 | 0.166 |
|  | Based on the median | 0.405 | 1 | 201 | 0.525 |
|  | Based on the median and with adjusted gl | 0.405 | 1 | 200,830 | 0.525 |
|  | Based on the trimmed mean | 1.929 | 1 | 201 | 0.166 |
| Headache | Based on the average | 2.955 | 1 | 201 | 0.087 |
|  | Based on the median | 0.815 | 1 | 201 | 0.368 |
|  | Based on the median and with adjusted gl | 0.815 | 1 | 198,048 | 0.368 |
|  | Based on the trimmed mean | 2.955 | 1 | 201 | 0.087 |
| Difficulty concentrating | Based on the average | 0.178 | 1 | 201 | 0.673 |
|  | Based on the median | 0.045 | 1 | 201 | 0.832 |
|  | Based on the median and with adjusted gl | 0.045 | 1 | 200.506 | 0.832 |
|  | Based on the trimmed mean | 0.178 | 1 | 201 | 0.673 |
| Loss of taste | Based on the average | 12.109 | 1 | 201 | <0.001 |
|  | Based on the median | 1.786 | 1 | 201 | 0.183 |
|  | Based on the median and with adjusted gl | 1.786 | 1 | 200.773 | 0.183 |
|  | Based on the trimmed mean | 12.109 | 1 | 201 | <0.001 |
| Loss of smell | Based on the average | 0.813 | 1 | 201 | 0.368 |
|  | Based on the median | 0.154 | 1 | 201 | 0.695 |
|  | Based on the median and with adjusted gl | 0.154 | 1 | 201.000 | 0.695 |
|  | Based on the trimmed mean | 0.813 | 1 | 201 | 0.368 |
| Mood disturbance | Based on the average | 3.474 | 1 | 201 | 0.064 |
|  | Based on the median | 0.779 | 1 | 201 | 0.378 |
|  | Based on the median and with adjusted gl | 0.779 | 1 | 201.000 | 0.378 |
|  | Based on the trimmed mean | 3.474 | 1 | 201 | 0.064 |
| Palpitations | Based on the average | 3.436 | 1 | 201 | 0.065 |
|  | Based on the median | 1.015 | 1 | 201 | 0.315 |
|  | Based on the median and with adjusted gl | 1.015 | 1 | 199.167 | 0.315 |
|  | Based on the trimmed mean | 3.436 | 1 | 201 | 0.065 |
| Difficulty swallowing | Based on the average | 0.061 | 1 | 201 | 0.806 |
|  | Based on the median | 0.014 | 1 | 201 | 0.906 |
|  | Based on the median and with adjusted gl | 0.014 | 1 | 200.995 | 0.906 |
|  | Based on the trimmed mean | 0.061 | 1 | 201 | 0.806 |
| Conjunctivitis | Based on the average | 4.137 | 1 | 201 | 0.043 |
|  | Based on the median | 2.180 | 1 | 201 | 0.141 |
|  | Based on the median and with adjusted gl | 2.180 | 1 | 200.449 | 0.141 |
|  | Based on the trimmed mean | 4.137 | 1 | 201 | 0.043 |
